# Color and Chemical Stability of 3D-Printed and Thermoformed Polyurethane-Based Aligners

**DOI:** 10.3390/polym16081067

**Published:** 2024-04-11

**Authors:** Luka Šimunović, Sara Čekalović Agović, Antun Jakob Marić, Ivana Bačić, Eva Klarić, Flavio Uribe, Senka Meštrović

**Affiliations:** 1 Department of Orthodontics, School of Dental Medicine, University of Zagreb, 10000 Zagreb, Croatia; lsimunovic@sfzg.hr; 2 School of Dental Medicine, University of Zagreb, 10000 Zagreb, Croatia; scekalovic@sfzg.hr; 3 Faculty of Mechanical Engineering and Naval Architecture, University of Zagreb, 10000 Zagreb, Croatia; antun@3dtech.hr; 4 Forensic Science Centre “Ivan Vučetić”, Ministry of the Interior, 10000 Zagreb, Croatia; ivana.bacic@mup.hr; 5 Department of Endodontics and Restorative Dentistry, School of Dental Medicine, University of Zagreb, 10000 Zagreb, Croatia; eklaric@sfzg.hr; 6 Department of Orthodontics, School of Dental Medicine, University of Connecticut, Farmington, CT 06030, USA; furibe@uchc.edu

**Keywords:** aligners, 3D printing, color, Fourier transform infrared spectroscopy

## Abstract

The significant rise in the use of clear aligners for orthodontic treatment is attributed to their aesthetic appeal, enhancing patient appearance and self-confidence. The aim of this study is to evaluate the aligners’ response to common staining agents (coffee, black tea, Coca-Cola, and Red Bull) in color and chemical stability. Polyurethane-based thermoformed and 3D-printed aligners from four brands were exposed to common beverages to assess color change using a VITA Easyshade compact colorimeter after 24 h, 48 h, 72 h, and 7 days, as well as chemical stability using ATR-FTIR spectroscopy. The brand, beverage, and manufacturing method significantly influence color stability. ATR-FTIR analysis revealed compositional differences, with variations in response to beverage exposure affecting the integrity of polymer bonds. Color change analysis showed coffee as the most potent staining agent, particularly affecting Tera Harz TC85 aligners, while ClearCorrect aligners exhibited the least susceptibility. 3D-printed aligners showed a greater color change compared to thermoformed ones. Aligners with a PETG outer layer are more resistant to stains and chemical alterations than those made of polyurethane. Additionally, 3D-printed polyurethane aligners stain more than thermoformed ones. Therefore, PETG-layered aligners are a more reliable choice for maintaining the aesthetic integrity of aligners.

## 1. Introduction

The utilization of clear aligners in orthodontic treatment has significantly increased over the past two decades, driven by the growing demand for aesthetically pleasing orthodontic solutions [1,2,3]. Aligners, lauded for their aesthetic superiority over conventional braces, have become a preferred choice for patients who seek an inconspicuous method for correcting malocclusions [4,5]. The appeal of these aligners lies in their transparency, which allows patients to undergo orthodontic treatment without the stigma often associated with metal braces, thereby not only enhancing the patient’s appearance during treatment but also boosting self-confidence [6].

The manufacturing of clear aligners can be broadly classified into thermoformed and 3D-printed techniques. Thermoforming involves heating a polymer sheet until it becomes pliable and then molding it over a dental model to form the aligner [7]. Those are composed of thermoplastic resin polymers such as poly(vinyl chloride) (PVC), poly(ethylene terephthalate) (PET), poly(ethylene terephthalate glycol) (PETG), and thermoplastic polyurethane (TPU). On the other hand, 3D printing, or additive manufacturing, builds the aligner layer by layer directly from a digital model [8,9,10]. These methods employ polyurethane-based materials, chosen for their balance of clarity, strength, and flexibility, which are essential for the continuous force application required to move teeth into their desired positions [9,11]. Polyurethane-based materials, in particular, have been identified for their superior performance in terms of mechanical properties and patient comfort [11,12].

A novel approach in 3D printing utilizes polyfunctional acrylic non-isocyanate hydroxyurethanes, offering an innovative route for creating photocurable thermoset resins suitable for applications like stereolithography. This method bypasses the need for isocyanates, known for their moisture sensitivity, enabling more resilient and adaptable dental aligners through photo cross-linking techniques [13]. Additionally, research into thermosetting nonlinear optical polymers such as polyurethane showcases the potential for creating materials that undergo thermal curing post-electric field poling, indicating a route for enhancing the mechanical properties and long-term stability of aligners through controlled cross-linking [14]. The cross-linking extent and polymerization techniques, especially in 3D-printed polyurethane aligners, focus on achieving a balance between printability before curing and robustness after final thermosetting. Techniques have been developed to enable the thermoset polymers’ shaping without cross-linking or excessive fillers, leading to innovations in creating complex 3D structures with isotropic mechanical properties, thereby overcoming traditional limitations of thermosetting resins in 3D-printing processes [15]. The chemistry underlying these advancements encompasses both the meticulous design of polymer networks and the strategic application of polymerization and cross-linking methods to yield dental aligners that are not only effective in treatment but also superior in material properties and comfort.

The aspect of color stability in aligners is paramount, as any discoloration can significantly undermine their aesthetic value. Previous studies on thermoformed aligners have extensively explored their resistance to staining, attributing their color stability to material properties and manufacturing processes [16,17]. Research has shown that thermoformed aligners retain their color when exposed to common dietary agents such as coffee, tea, and wine, primarily due to the surface characteristics and the chemical composition of the polyurethane material used [18].

The chemical composition of dental materials, such as aligners and restoratives, is significantly influenced by exposure to common beverages, leading to changes in their physical and optical properties [19]. Acidic beverages can cause erosion, deteriorate material surfaces, and make them prone to staining and structural weakening [20]. Staining agents like coffee and tea contain chromogenic compounds that adhere to or penetrate these materials, leading to discoloration [21]. Additionally, water absorption from beverages contributes to the hydrolytic degradation of polymers, affecting their mechanical properties and aesthetic appeal [19]. Beverages also contain additives, including acids and colorants, that can chemically interact with dental materials, further exacerbating degradation and staining [20]. Both smoke and beverages can impact the color stability of dental appliances, but the presence of smoke specifically exacerbates discoloration and deterioration, posing a significant threat to both the aesthetic and functional integrity of the appliances [21]. Moreover, the temperature of beverages can induce thermal expansion or contraction in these materials, increasing their susceptibility to damage and discoloration over time [22].

However, with the advent of 3D-printing technology in the fabrication of aligners, there is a compelling need to examine whether these aligners exhibit the same level of color stability as their thermoformed counterparts. Three-dimensional printing offers several advantages, including the ability to produce aligners with complex geometries and a customized fit, but its impact on the color stability of the final product remains under-investigated [23]. Given the different processing conditions and material formulations involved in 3D printing, it is critical to understand how these factors influence the optical properties of the aligners over time [24]. A few preliminary studies have begun to explore this, suggesting that while 3D-printed aligners show promise in terms of fit and comfort, their resistance to staining and color change under various environmental conditions warrants further investigation [22,25,26].

In light of this, the present study seeks to delve deeper into the color stability of 3D-printed aligners, comparing their performance against the well-documented color stability of thermoformed aligners. By evaluating the aligners’ response to common staining agents, this research aims to offer comprehensive insights into the long-term aesthetic durability of 3D-printed aligners, thus filling a significant gap in orthodontic research and potentially guiding future material and process development for aligner fabrication.

## 2. Materials and Methods

### 2.1. Sample Preparation

Four polyurethane-based aligners from two brands of thermoformed aligners, ClearCorrect (Straumann, Basel, Switzerland) and Invisalign (Align Technology, San Jose, CA, USA), as well as two brands of 3D-printed aligners, Tera Harz TC-85 resin (Graphy, Seoul, Republic of Korea) and Clear-A (Senertek, İzmir, Turkey), were used in the study, one for each beverage (Coca-Cola, black coffee, black tea, and Red Bull). Ten composite Gradia Direct Anterior (GC, Tokyo, Japan) A2 shade tooth models, teeth 15–25, were made for each aligner. The most common tooth shade among individuals aged 20–40, who are the primary demographic for aligner therapy, is the A2 shade. This finding is supported by a study that evaluated tooth shade among a group of patients and found that A2 was among the most common shades, indicating its prevalence in a broad population range [27,28].

### 2.2. Beverage Preparation

Use of standard commercial brands of Coca-Cola (Coca-Cola HBC Hrvatska, Zagreb, Croatia), black coffee (Franck jubilarna original, Franck d.d., Zagreb, Croatia), black tea (Franck d.d., Zagreb, Croatia), and Red Bull (Red Bull GmbH, Fuschl am See, Austria). A tea filter bag was added to 2 dL of hot water (90 °C) and brewed for 3 min, while the coffee was prepared as following: 2 full teaspoons of coffee was added to 1 dL of boiling water, mixed, and heated again gently until the foam rose. The beverages were left to cool down at room temperature. The samples were stored in a Cultura incubator (Ivoclar Vivadent, Schaan, Liechtenstein) at a temperature of 37 °C. To compensate for the loss due to evaporation, the solutions in which the samples were immersed were refreshed every 24 h throughout the experiment.

### 2.3. Color Change Evaluation

A standard VITA Easyshade compact colorimeter was used to check the color change, which was evaluated at 5 intervals: T0 (before immersing into the solution), T_1_ after 24 h, T_2_ after 48 h, T_3_ after 72 h, and T_4_ after 7 days. All the measurements were taken in the same room with a standardized light source.

The color change was measured following the Commission Internationale de I’Eclairage L*a*b* color system. The color parameter L* represents the lightness (+ is lighter, − is darker), a* is the red/green coordinate (+ is redder, − is greener), and b* is the yellow/blue coordinate (+ is yellower, − is bluer). The total color change (ΔE*) [Δ*E* = [(Δ*L*)^2^ + (Δ*a*)^2^ + (Δ*b*)^2^]^½^] value between T_0_ and T_1_ was calculated using the formula (ΔE_T0−T1_)^2^ = (L_T1_ − L_T0_)^2^ + (a_T1_ − a_T0_)^2^ + (b_T1_ − b_T0_)^2^ and was similarly calculated between T_2_ and T_0_, T_3_ and T_0_, and T_4_ and T_0_.

**Figure 1:**
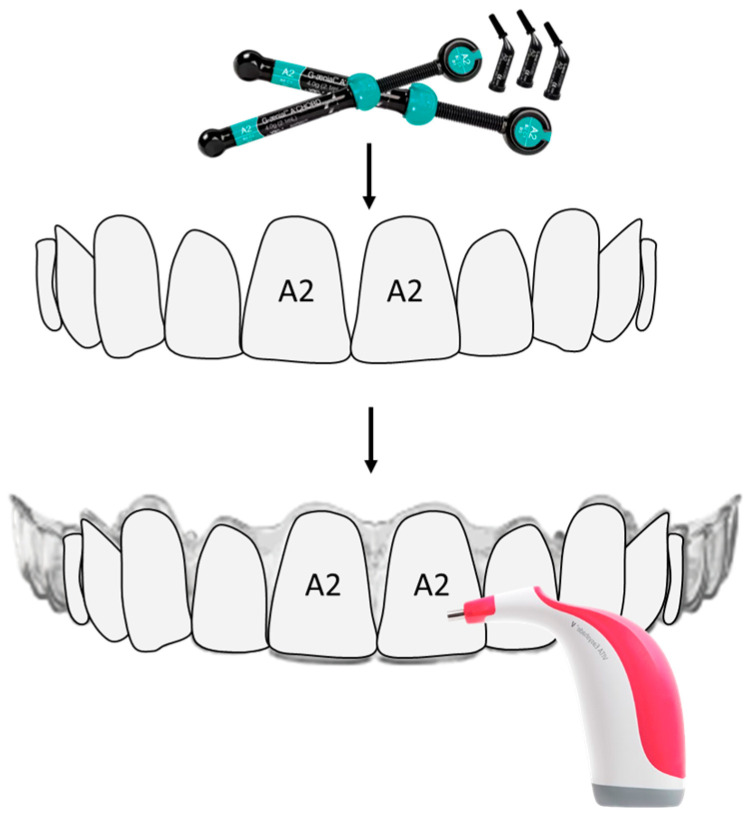
Scheme of teeth models and measurement.

Standard measurements were performed by an investigator who was blind to the group division. The flat labial surface of upper tooth 15 to tooth 25 of each aligner was measured. A tooth model was made using composite resin Gradia Direct Anterior (GC, Tokyo, Japan) with an A2 shade in an aligner template isolated with glycerin (Vazelin, Balea, dm-drogerie markt, Karlsruhe, Germany). These models were used as the background reference and set behind the labial surface of each aligner (Figure 1).

### 2.4. Color Change Rating

The color change rating was determined with the help of the National Bureau of Standards (NBS) system to express color differences [29]. The ΔE* value was converted into NBS units with the formula NBS = ΔE* × 0.92 to relate the magnitude of color change to the clinical relevance standard [30]. NBS rating values are as follows: 0.1–0.5, extremely slight change; 0.5–1.5, slight change; 1.5–3.0, perceivable change; 3.0–6.0, marked changes; 6.0–12.0, extremely marked change; and 12.0 or more, change to another color.

### 2.5. ATR-FTIR

Fourier transform infrared spectroscopy (FTIR) spectra in the 4000–400 cm^−1^ range were collected using a Bruker Alpha FTIR spectrometer (Bruker Optics, Ettlingen, Germany) with an ATR accessory. Spectra are the results of 10 continuous scans at a resolution of 4 cm^−1^ [11]. For instrument control and spectra manipulation, OPUS v7.0 software was used.

### 2.6. Sample Size

The a priori required sample size was 36 for eff. size 0.25, α err prob 0.05, power (1-β err prob) 0.8 with 4 groups and 4 points of measurement. So, 9 measurements were taken per period, per aligner, and per beverage. To be sure of achieving power, we measured a color change of 10 points on each aligner, in the middle of the labial surface of teeth 15–25. This calculation aligns with the recommendation for ensuring sufficient statistical power to detect meaningful effects in clinical and experimental research, especially in fields requiring precise outcome measurements from multiple groups over various time points [31].

### 2.7. Statistical Analysis

Statistical analyses were performed using IBM SPSS Statistics software, version 29.0.1.0 (IBM, New York, NY, USA) to assess the impact of beverage type, brand, and manufacturing method on the color change and stability of the tested materials. Descriptive statistics, including means and standard deviations, are presented to summarize the data. This comprehensive analysis included both within-subjects and between-subjects effects, aiming to uncover significant interactions and trends. A Box’s Test was employed to check for equal covariances, a prerequisite for a general linear model (GLM). Moreover, the influence of the independent variables and their interplay was examined through multivariate tests, with Pillai’s trace utilized to determine significance. The assumption of sphericity, crucial for a repeated measures ANOVA, was tested using a Mauchly’s test. Any detected violations were corrected using the Greenhouse–Geisser adjustment. The threshold for statistical significance was set at *p* < 0.05.

## 3. Results

### 3.1. ATR-FTIR

**Figure 2:**
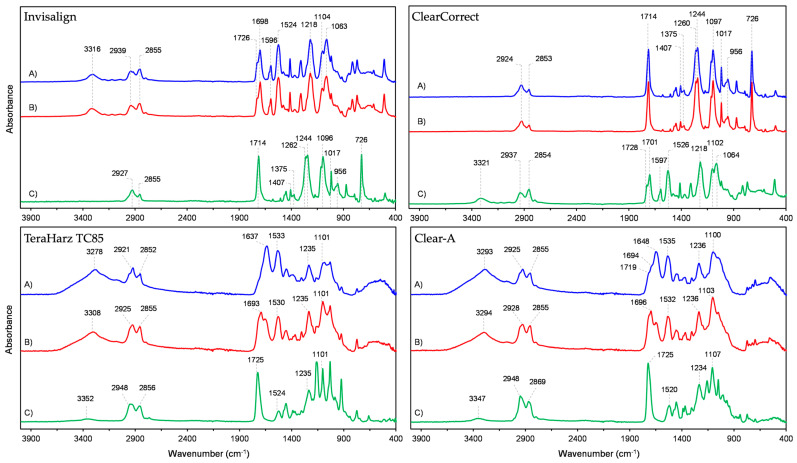
Material characterization: A—outer vestibular layer, B—outer layer in contact with teeth, C—middle layer.

The ATR-FTIR method was performed for the compositional characterization of polymeric thermoformed and 3D-printed aligners before and after exposure to different beverages. The representative spectra of the control aligner samples of each brand are shown in Figure 2. Specifically, spectra were recorded from different areas of each aligner, including the incisor, canine, and molar regions. According to the FTIR spectra, Invisalign and Clear Correct are three-layer aligners both made of polymeric materials based on poly(ethylene terephthalate glycol) (PETG) and polyurethane (PU), but with a different layer sequence. The layer arrangement for Invisalign can be shown schematically as PU–PETG–PU, while Clear Correct has the order PETG–PU–PETG (Figure 2). The mentioned materials are identified based on their characteristic peaks, and a detailed assignment of their FTIR spectra was reported in previous studies [32,33], while the identification of the FTIR peaks for the Invisalign samples is presented in Table 1.

Aligners from TeraHarz TC85 and Clear-A, are made only of polyurethane, and even though these are single-layer materials, FTIR analysis revealed significant variations in the spectra depending on the position from which the samples were extracted. This variability can be attributed to the type of material since a similar anomaly, although to a lesser extent, was also found on the outer polyurethane layers of the Invisalign aligner.

Analysis of the outer layer of samples exposed to different beverages showed that the least changes were observed for the ClearCorrect aligner. The exposure of this material to tea, coffee, and Red Bull did not cause any changes in the PETG spectrum compared to the control sample, while changes in the spectrum of the sample exposed to Coca-Cola are manifested through the appearance of a weak band at 1534 cm^−1^ and a broad and weak band in the region of the stretching vibration of N-H or O-H bonds.

**Figure 3:**
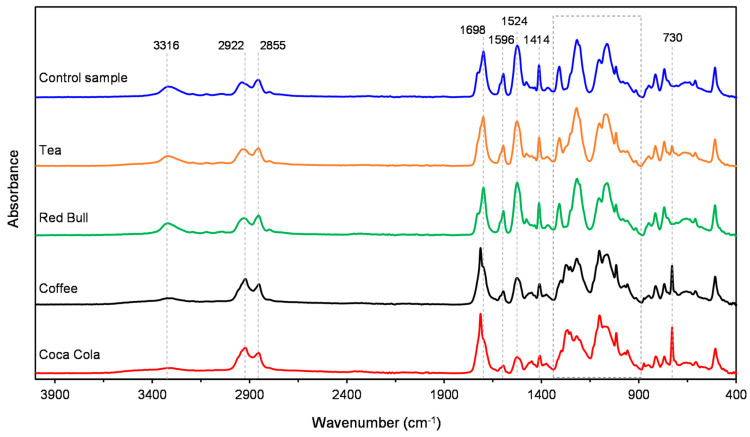
The spectra of the vestibular outer layer of the control sample of the Invisalign aligner as well as the samples treated with the selected beverages.

The spectra of the outer layer of the control sample of the Invisalign aligner as well as the samples treated with the selected beverages are shown in Figure 3.

It was determined that the spectra of the control sample and the sample exposed to Red Bull did not differ, while the spectrum of the sample kept in tea showed a weak band at 730 cm^−1^. On the other hand, the spectrum profiles of samples exposed to coffee and Coca-Cola show significant deviations from the control sample, which are manifested through the development of an intense band at 730 cm^−1^, the appearance of new peaks, and their low resolution in the area of the vibrational modes of C–O–C bonds as a part of polyurethane ester linkage (~1350–1000 cm^−1^) as well as a decrease in the intensity of the bands at 1596 cm^−1^ and 1527 cm^−1^ which originate from C=C stretching vibrations in the aromatic ring and bending of the N-H group, respectively. In these samples, a weaker influence of hydrogen bonding was also observed, which is reflected as an increase in the intensity of the non-hydrogen-bonded carbonyl band at 1714 cm^−1^ and the appearance of a broad band of non-hydrogen-bonded N–H stretching vibrations in the range 3560–3400 cm^−1^, with a simultaneous decrease in the intensity of the bands of their hydrogen-bonded forms.

The spectra of the printed TeraHarz 85 and Clear-A aligners exposed to beverages as well as their control samples showed great variability and therefore cannot be compared. The spectra of the treated samples do not contain any bands originating from the used beverages. The observed changes in the spectra are most likely due to uneven polymerization.

### 3.2. Color Change

Our analysis unveiled notable disparities in color change, driven by brand, beverage, and manufacturing method. The mean and standard deviation for each group combination are detailed for time points ΔE* (T_0_ − T_1_), ΔE* (T_0_ − T_2_), ΔE* (T_0_ − T_3_), and ΔE* (T_0_ − T_4_), and are presented in Table 2. Coffee emerged as the most impactful beverage, inducing the highest color change (mean = 15.156), notably more than the other beverages such as black tea (mean = 9.052) and Coca-Cola and Red Bull (means = 4.374 and 4.584, respectively), with each comparison yielding significant differences (*p* < 0.001). Brand-wise, Tera Harz TC85 aligners demonstrated the highest susceptibility to color change, contrasting with ClearCorrect aligners, which were the least affected.

A Box’s Test revealed a significant variability in color change across groups (Box’s M = 477.500, F (150, 13,991.806) = 2.611, *p* < 0.001), indicating a violation of the GLM’s equal covariances assumption. Multivariate tests showed a significant effect of beverage type on color change (Pillai’s trace = 0.692, F (3, 142) = 106.486, *p* < 0.001), with interactions between beverage and brand (Pillai’s trace = 0.291, F (18, 432) = 2.579, *p* < 0.001) and among beverage, brand, and manufacturing method (Pillai’s trace = 0.844, F (9, 432) = 18.796, *p* < 0.001), highlighting the brand-dependent impact of beverages on color stability.

The Mauchly’s test indicated sphericity violation (W = 0.846, χ^2^(5) = 23.818, *p* < 0.001), leading to the Greenhouse–Geisser correction. This analysis found significant within-subjects effects of ΔE* (F (2.709, 390.059) = 152.349, *p* < 0.001, partial eta squared (η_p_^2^) = 0.514), demonstrating the treatment conditions’ influence on color stability. The interaction of ΔE* with the brand was significant (F (5.417, 390.059) = 10.965, *p* < 0.001, η_p_^2^ = 0.132), showing brand variability in color change. Additionally, linear trends in ΔE*s’ interactions with manufacturing method, brand, and beverage were significant (F (1) = 320.994, *p* < 0.001, η_p_^2^ = 0.690; F (2) = 18.218, *p* < 0.001, η_p_^2^ = 0.202), indicating a strong linear trend in color change and brand-specific stability differences.

Between-subjects effects analysis revealed a significant baseline difference in color change (F (1, 144) = 3826.988, *p* < 0.001), with significant impacts of the brand (F (2, 144) = 54.838, *p* < 0.001) and beverage type (F (3, 144) = 356.181, *p* < 0.001). The interaction between brand and beverage type also showed significant differences in color stability outcomes (F (6, 144) = 40.396, *p* < 0.001), underscoring the combined effect of these factors on aligner color stability.

#### 3.2.1. Manufacturing Method

The manufacturing method significantly affects the color stability of aligners. Thermoformed aligners result in a lower mean color change (5.208) compared to 3D-printed aligners (11.376), with a mean difference of 6.168 (*p* < 0.001). This substantial difference in color change between the two methods was also reflected in the grand mean color change (8.292) for all samples. These results strongly suggest that the type of manufacturing method plays a crucial role in the color stability of aligners.

#### 3.2.2. Brand

Further analysis revealed brand-specific impacts, with Tera Harz TC85 showing the greatest mean color change, significantly differing from ClearCorrect, which exhibited the lowest change (*p* < 0.001 across all brand comparisons). The mean differences between brands are presented in Table 3. The univariate tests for brand effect have a high η_p_^2^ value (0.816), suggesting a strong association.

#### 3.2.3. Beverage

Beverage impact was further corroborated by significant mean differences in pairwise comparisons, especially between coffee and the other beverages (*p* < 0.001), illustrating a strong association between beverage type and color change (F (3, 144) = 356.181, *p* < 0.001, η_p_^2^ = 0.881) (Table 4).

#### 3.2.4. Repeated Measures of Color Change ΔE*

The data present a clear trend in color change (ΔE*) over the four time points. There is a progressive increase in mean color change from time points 1 to 4, with significant differences between each consecutive time point (all *p* < 0.001 after Bonferroni adjustment). The multivariate tests corroborate these findings with high effect sizes (η_p_^2^ = 0.692) and statistical power, indicating a strong time effect on color change.

#### 3.2.5. L*a*b Ratios in Color Change

The effect of beverages on L*a*b parameters showed the following:

Coca-Cola significantly had a higher L* ratio compared to coffee (mean difference = 0.12742, *p* = 0.009), while black tea had a significantly higher a ratio compared to coffee (mean difference = 0.06502, *p* = 0.003) and Red Bull (mean difference = 0.09164, *p* < 0.001), suggesting black tea caused more red/green coloration. Coffee had a significantly higher b ratio compared to black tea (mean difference = 0.14146, *p* = 0.002) and Coca-Cola (mean difference = 0.16363, *p* < 0.001), suggesting that coffee exposure led to a greater yellow/blue coloration. Table 5 presents the contribution of each parameter (L*, a*, and b*) to the cumulative color change (ΔE*) of aligners from four brands when exposed to different beverages.

#### 3.2.6. Color Change Rating

Using the National Bureau of Standards (NBS) system to quantify color changes, the data revealed that coffee was the most potent staining agent across all brands, producing the highest NBS values, indicative of ‘extremely marked changes’ in color (Table 6). In particular, Tera Harz TC85 aligners were most affected by both coffee and black tea, showing NBS values that suggest drastic color transformations. Invisalign and ClearCorrect aligners were relatively more resistant, although coffee still resulted in ‘marked changes’ for ClearCorrect and ‘extremely marked changes’ for Invisalign. Black tea, Coca-Cola, and Red Bull also caused noticeable color changes across brands, with the impact of Red Bull ranging from ‘perceivable’ to ‘marked changes’.

**Figure 4:**
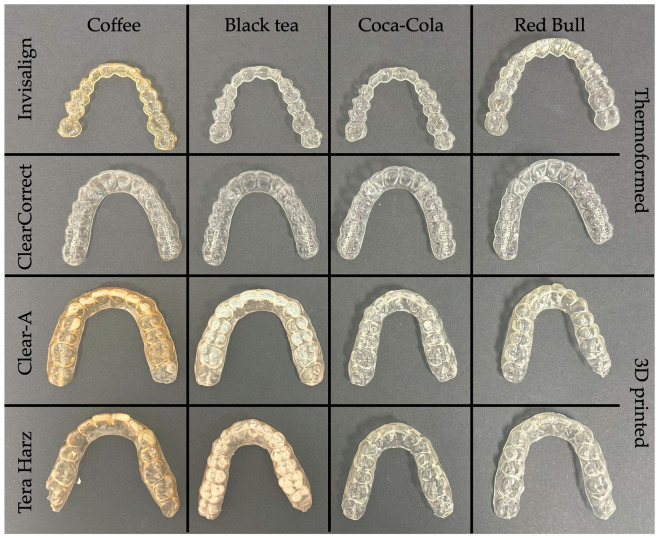
Aligners after 24 h of immersion.

**Figure 5:**
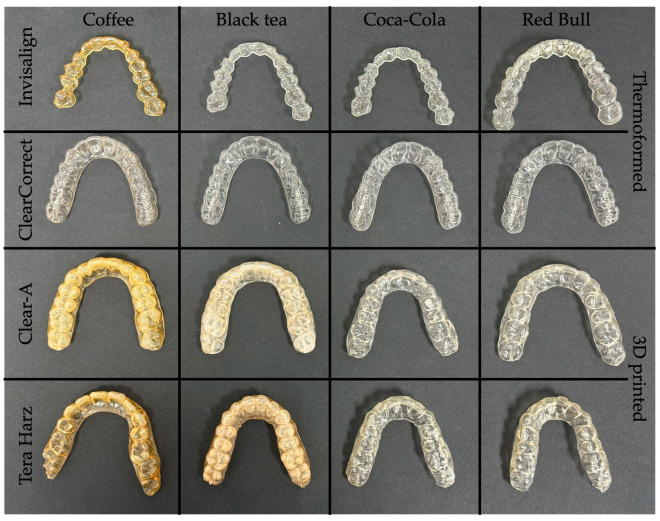
Aligners after 48 h of immersion.

**Figure 6:**
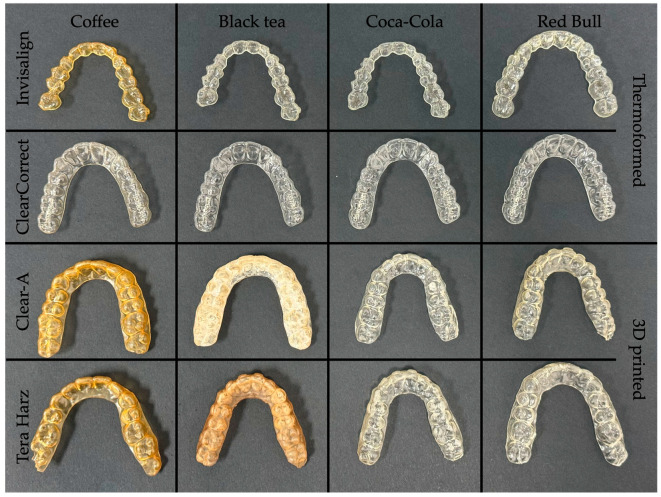
Aligners after 72 h of immersion.

**Figure 7:**
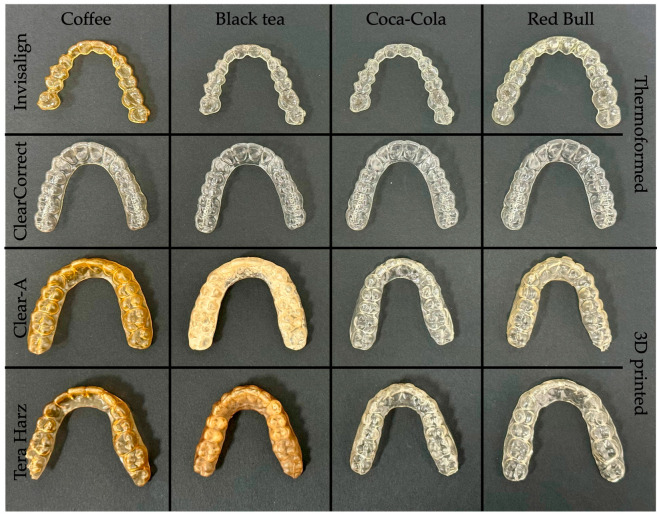
Aligners after 7 days of immersion.

Figure 4, Figure 5, Figure 6 and Figure 7 show aligners after 24, 48, 72 h, and 7 days of immersion. Three-dimensional-printed aligners experienced a drastic color transformation to a different color when exposed to coffee, with this extreme change occurring within just 24 h. Invisalign aligners, while more resistant, still reached this level of color change after 48 h of coffee immersion. Notably, ClearCorrect aligners showcased remarkable resistance, with no such extreme color change observed even after 7 days in coffee.

When considering the effects of black tea, Tera Harz TC85 aligners were notably susceptible, undergoing an extreme color change after 48 h. Clear-A aligners followed, with a similar level of color change noted at 78 h of exposure. Thermoformed aligners, in contrast, displayed superior resistance, showing no extreme color change even after 7 days of immersion in black tea.

## 4. Discussion

Color stability is essential for the aesthetic appeal and patient satisfaction of orthodontic aligners. To achieve this, clear aligner materials must be exceptional at transmitting light, ideally allowing more than 80% of visible light to pass through for maximum clarity. The materials of choice for these aligners are amorphous thermoplastic polymers, valued for their high translucency compared to the visually less-appealing, opaque crystalline polymers. Polymers such as polyurethane, polyester, poly(vinyl chloride), polysulfone, and polycarbonate are particularly favored for their beneficial optical properties [16]. Clear aligners’ consistent transparency and aesthetic appeal are crucial for their popularity [16]. Despite challenges like discoloration from consuming colored beverages, UV light exposure, and mouthwash use, these aligners are designed to maintain their clarity for one to two weeks of oral use, ensuring they meet the demands for both appearance and functionality [34]. Despite medical advice to remove aligners before eating or drinking anything other than water to prevent staining, research shows that a significant number of patients disregard these guidelines. They continue to eat and drink with their aligners on, compromising their transparency and, as a result, their aesthetic appearance. In fact, one study found that nearly half of all patients chose not to remove their aligners when consuming food and beverages [16,34].

Research has explored how clear aligners, made from thermoplastic polyurethane, PETG, ester copolymers, and PET, respond to staining substances [35,36,37]. However, findings on 3D-printed aligners are lacking.

Our analysis highlighted significant variations in color change, influenced by the brand of the aligner, the type of beverage and the manufacturing process used. The method used to manufacture aligners significantly influences their color stability, with thermoformed aligners exhibiting less susceptibility to color changes compared to those made using 3D-printing technologies. Thermoforming, a process where a plastic sheet is heated to a pliable forming temperature, formed to a specific shape in a mold, and then trimmed to create a usable product, tends to preserve better the original color integrity of the material [38,39]. This preservation is attributed to the uniform material distribution achieved during the thermoforming process, which could minimize the exposure of the polymers to conditions that could predispose them to discoloration. In contrast, 3D printing, which involves the layer-by-layer addition of material to build the final product, might introduce micro-porosities or variations in the material that increase its propensity to absorb pigments from food, drinks, and other external agents, leading to a higher degree of color change over time [8,22,40].

Furthermore, 3D-printed polyurethane materials may exhibit more staining compared to their thermoformed counterparts due to differences in surface characteristics and material properties arising from their respective manufacturing processes. Specifically, 3D printing often results in parts with higher surface roughness and porosity, which could trap staining agents more easily, whereas thermoforming tends to produce parts with smoother and denser structures that are less prone to staining [41]. The formulations of polyurethane used in 3D printing might also differ from those in thermoforming, with additives in 3D-printing materials potentially affecting stain resistance. Additionally, the thermal history and microstructure from the manufacturing processes could influence the material’s stain resistance, with 3D-printed materials potentially having more reactive sites for staining due to rapid cooling and layer-by-layer construction. Furthermore, chemical exposure during 3D printing could alter surface properties, impacting stain interaction [42,43,44].

Further scrutiny into the effects of different brands on the color stability of orthodontic aligners has brought to light distinct disparities in how various materials react to potential staining agents. In this analysis, it was discovered that aligners made from Tera Harz TC-85 resin underwent the most substantial mean color change when exposed to staining substances compared to Clear-A, Invisalign, and ClearCorrect. This finding starkly contrasts with the performance of ClearCorrect aligners, which demonstrated minimal color alteration among the brands tested. Various studies have concluded that polyurethane is more susceptible to pigment adsorption and does not provide adequate color stability [36]. This significant variation underscores the influence of material composition and the proprietary manufacturing processes employed by each brand on the aligners’ susceptibility to discoloration.

The gradual escalation in the average color change of orthodontic aligners across different time intervals, demonstrating a steady progression in discoloration, is supported by the findings of several studies. For instance, Liu et al. [16] evaluated the color stabilities of three types of orthodontic clear aligners exposed to staining agents and observed slight color changes after short-term exposure, with significant differences in color change (ΔE*) after longer exposures, indicating a continuous and measurable deterioration in the aligners’ appearance over time.

Furthermore, Venkatasubramanian et al. [30] conducted an in vitro study examining how clear aligners changed color upon exposure to various indigenous food products. The study found that the hue of the aligners noticeably changed when exposed to substances like turmeric, saffron, Kashmiri red chili powder, and coffee at both 12 and 24 h intervals, reinforcing the trend of worsening color stability over time.

This study’s findings regarding the impact of beverages on the color stability of orthodontic aligners are supported by existing research, which indicates significant variances in how different beverages affect aligner materials. Coffee, in particular, has been identified as a major culprit in inducing color change across various types of orthodontic appliances and materials, such as aesthetic ceramic brackets, adhesive samples, and aligner materials [34,45]. Moreover, Liu et al. [16] investigated the color stability of three types of orthodontic clear aligners exposed to staining agents, including coffee. They found that Invisalign aligners stained with coffee exhibited significantly higher color changes compared to other beverages, highlighting the beverage-specific impact on aligner aesthetics, particularly the detrimental effect of coffee, which is confirmed in our study.

The L*a*b color system is designed to encompass all perceivable colors, where ‘L*’ represents lightness, ‘a*’ denotes the spectrum from green to red, and ‘b*’ captures the spectrum from blue to yellow. Through this analysis, it was observed that exposure to black tea resulted in a noticeable shift toward redness in the aligners, as indicated by an increase in the ‘a*’ value. This suggests that compounds in black tea, such as tannins, have a specific effect on the aligner material that accentuates red hues [34]. Conversely, the impact of coffee on aligners was distinctly different, leading to an increase in the ‘b*’ value, which signifies a shift toward more yellow tones. The yellowing effect caused by coffee can be attributed to the presence of chromogens and other staining molecules in coffee that have a strong affinity for the aligner material, embedding within and altering its intrinsic color to a more yellow shade [16]. Considering that the FTIR spectra of the treated samples in this study do not contain any bands originating from the used beverages, an additional explanation could be that PU materials are susceptible to yellowing when exposed to ultraviolet (UV) radiation and oxygen, a phenomenon attributed to the presence of nitrogen atoms within their structure. This yellowing process is a result of photochemical degradation, which involves the scission of the urethane group and photooxidation of the central methylene group situated between aromatic rings. These reactions lead to the formation of quinone structures, which are yellow chromophoric reaction products, thus causing the PU surface to yellow. The process is quantifiable by measuring changes in the CIELab* color components, where a systematic tendency toward higher values with increasing irradiation time is observed, indicating a greater degree of yellowing. This degradation is correlated with an increase in carbonyl group concentration, further evidencing the chemical changes occurring within the PU material under UV exposure [46].

The CIELab* color system is widely endorsed for assessing color changes in dentistry, as it reflects human perception [47]. It is generally agreed among researchers that color alterations with a ΔE* value of 3.7 or higher, as measured by a spectrophotometer, are noticeable to the naked eye or clinically unacceptable [48,49]. Therefore, in this study, any color change values below 3.7 were deemed satisfactory, which was present only in Invisalign after 7 days of immersion in Coca-Cola, and ClearCorrect in black tea and Coca-Cola.

To address the staining issues caused by beverages on orthodontic aligners, various cleaning methods have been investigated for their effectiveness and impact on the thermoplastic materials used in aligners [50,51,52,53]. Mechanical brushing, often recommended for daily hygiene, can remove surface stains but must be performed gently to avoid micro-scratches that could harbor bacteria and increase staining over time. Chemical cleaners, such as hydrogen peroxide-based solutions or specialized orthodontic cleaning tablets, offer an alternative that can reduce staining without physical abrasion. Studies have shown that these chemical agents can effectively minimize discoloration without significantly altering the mechanical properties of the aligners, such as tensile strength or elasticity. However, the excessive use of harsh chemicals should be avoided as they may cause brittleness or unwanted changes in the aligner material over extended periods. Ultimately, the choice of cleaning method should balance effectiveness in stain removal with the preservation of the aligner’s integrity and comfort for the patient [50,51,52,53].

The significant changes observed in the spectrum profiles of polyurethane samples exposed to coffee and Coca-Cola, including the development of an intense band at 730 cm^−1^, the appearance of new peaks, and changes in the vibrational modes of C–O–C bonds as a part of polyurethane ester linkage, indicate alterations in the material’s chemical structure due to exposure to these substances. These changes could lead to alterations in the physical and mechanical properties of the polyurethane, such as its flexibility, strength, and durability [54,55,56]. The decrease in the intensity of the bands associated with C=C stretching vibrations and the bending of the N-H group suggests a weakening of these chemical bonds, potentially leading to decreased material robustness [57,58].

A reduction in hydrogen bonding, as indicated by changes in the intensity of bands associated with non-hydrogen-bonded forms, could affect the material’s thermal stability and water resistance. Polyurethane’s resistance to environmental factors such as temperature and moisture is crucial for its performance in various applications, from medical devices to coatings and insulations [59,60,61,62].

In clinical settings, materials like polyurethane are often chosen for their specific properties, such as biocompatibility, strength, and durability. Changes in these properties due to chemical exposure could impact the safety and efficacy of medical devices made from polyurethane. For instance, alterations in the material’s chemical structure could potentially lead to increased degradation rates, affecting the longevity and performance of implanted devices or coatings used in medical applications [63,64].

While our study offers valuable insights into the color and chemical stability of polyurethane-based aligners exposed to common beverages, it also presents several limitations that warrant consideration. Our methodology focused on a static evaluation of beverage-induced staining without considering the mitigating effects of daily cleaning routines. This aspect limits the applicability of our findings to real-life scenarios where aligners are regularly cleaned by users, potentially influencing the degree of discoloration experienced. The reliance on the CIELab* color difference formula over CIEDE2000 in dental research, primarily driven by its historical acceptance and straightforward methodology for quantifying color differences, introduces a limitation in terms of accurately capturing the nuances of human color perception. While CIELab* offers simplicity and wide applicability, it may not always reflect the perceptual color differences as accurately as the more sophisticated CIEDE2000 formula, especially in scenarios where a high degree of color discrimination is required. Therefore, future work should aim to establish an evaluation framework that encompasses both advanced colorimetric assessments, as well as an analysis of surface roughness and mechanical characteristics, since they affect aesthetics and functional longevity.

## 5. Conclusions

This study highlights that the difference in performance was notable between manufacturing methods, with 3D-printed polyurethane aligners showing more significant staining than thermoformed ones. Such disparities underscore the importance of manufacturing techniques in determining the resilience of aligners to staining substances like coffee. Further, our findings reveal that aligners (ClearCorrect) incorporating an outer layer of PETG demonstrate superior resistance to staining and chemical alterations compared to those fabricated entirely from polyurethane, which are more vulnerable to damage. These findings highlight that aligners with a PETG outer layer could offer a more stable option for those seeking to maintain the aesthetic quality of their orthodontic appliances.

## Figures and Tables

**Table 1:** FTIR peak identification for the Invisalign samples.

Polyurethane Layers		Poly (Ethylene Terephthalate Glycol) Layer	
Wavenumber (cm^−1^)	Vibration	Wavenumber (cm^−1^)	Vibration
3316	*ν* N–H	2927	*ν_as_* C–H (CH_2_)
2939	*ν_as_* C–H (CH_2_)	2855	*ν_s_* C–H (CH_2_)
2855	*ν*_s_ C–H (CH_2_)	1714	*ν* C=O
1726	*ν* C=O (free)	1407	*δ* CH_2_
1698	*ν* C=O (hydrogen bonded)	1375	*δ*_wg_ CH_2_
1596	*ν* C=C (aromatic)	1262	*ν* (C=O)–O
1524	*δ* N–H, *ν* C–C, *ν* C–N	1244	
1218	*ν* C–O	1096	*ν*_s_ C–O
1104	*ν* C–O–C (of ester group)	1017	*δ*_ip_ C–H
1063		956	*δ* C–H (cyclohexylene ring)
		726	*δ*_oop_ C–H

*ν* stretching, *δ* bending, s symmetric, as asymmetric, oop out of plane, ip in plane, wg wagging.

**Table 2:** Descriptive statistics (mean and standard deviation (SD)) of ΔE* across 5 time points and among different beverages and brands.

Brand	Beverage	ΔE* (24 h)		ΔE* (48 h)		ΔE* (72 h)		ΔE* (7 Days)	
		Mean	SD	Mean	SD	Mean	SD	Mean	SD
Invisalign	Coffee	10.64	2.43	13.38	2.3	16.95	2.68	20.06	2.61
	Black tea	2.89	0.96	2.71	0.73	3.69	1.58	4.75	2.34
	Coca-Cola	2.16	0.97	3.55	1.84	3.19	1.1	3.42	1.5
	Red Bull	4.74	1.9	4.66	1.69	4.01	1.55	6.87	6.52
ClearCorrect	Coffee	2.51	1.06	3.28	0.66	4.27	0.94	4.28	1.46
	Black tea	2.86	1.62	2.94	0.94	3.19	0.88	2.99	1.07
	Coca-Cola	3.43	0.85	3.79	1.15	3.91	0.77	3.62	1.04
	Red Bull	3.18	1.47	5.27	1.9	4.6	1.97	4.86	2.18
Clear-A	Coffee	15.02	4.72	19.45	1.76	19.72	1.12	23.38	2.12
	Black tea	6.54	1.82	8.45	1.71	12.48	1.98	17	1.93
	Coca-Cola	5.24	2.72	5.1	2.6	6.06	2.61	4.36	2.93
	Red Bull	5.48	2.14	4.82	3.28	3.57	2.29	4.92	2.81
Tera Harz TC85	Coffee	17.14	4.76	22.48	2.44	23.56	1.72	26.38	2.39
	Black tea	9.77	2.77	15.46	2.65	22.06	2.1	27.04	1.58
	Coca-Cola	3.89	1.57	6	2.77	6.79	2.11	5.49	2.05
	Red Bull	4.3	2.59	4.74	2.33	3.6	2.38	3.73	1.92

**Table 3:** The mean differences in color change ΔE among brands.

Brand (I)	Brand (J)	Mean Difference (I–J)	95% CI Lower	95% CI Upper	*p*-Value
Invisalign	ClearCorrect	3.041	2.027	4.055	<0.001
	Clear-A	−3.371	−4.386	−2.357	<0.001
	Tera Harz TC85	−5.924	−6.938	−4.909	<0.001
ClearCorrect	Invisalign	−3.041	−4.055	−2.027	<0.001
	Clear-A	−6.413	−7.427	−5.398	<0.001
	Tera Harz TC85	−8.965	−9.979	−7.951	<0.001
Clear-A	Invisalign	3.371	2.357	4.386	<0.001
	ClearCorrect	6.413	5.398	7.427	<0.001
	Tera Harz TC85	−2.552	−3.566	−1.538	<0.001
Tera Harz TC85	Invisalign	5.924	4.909	6.938	<0.001
	ClearCorrect	8.965	7.951	9.979	<0.001
	Clear-A	2.552	1.538	3.566	<0.001

**Table 4:** The mean differences in color change ΔE among beverages.

Beverage (I)	Beverage (J)	Mean Difference (I–J)	95% CI Lower	95% CI Upper	*p*-Value
Coffee	Black tea	6.103	5.089	7.117	<0.001
	Coca-Cola	10.781	9.767	11.796	<0.001
	Red Bull	10.571	9.557	11.586	<0.001
Black tea	Coffee	−6.103	−7.117	−5.089	<0.001
	Coca-Cola	4.678	3.664	5.692	<0.001
	Red Bull	4.468	3.454	5.482	<0.001
Coca-Cola	Coffee	−10.781	−11.796	−9.767	<0.001
	Black tea	−4.678	−5.692	−3.664	<0.001
	Red Bull	−0.210	−1.224	0.804	1.000
Red Bull	Coffee	−10.571	−11.586	−9.557	<0.001
	Black tea	−4.468	−5.482	−3.454	<0.001
	Coca-Cola	0.210	−0.804	1.224	1.000

**Table 5:** The contribution of each parameter (L*, a*, and b*) to the cumulative color change (ΔE*) of aligners from four brands exposed to different beverages.

Brand	Beverage	L* Parameter		a* Parameter		b* Parameter		ΔE*	
		Mean	SD	Mean	SD	Mean	SD	Mean	SD
Invisalign	Coffee	0.26 *	0.04	0.06 *	0.01	**0.68**	0.04	20.06	2.61
	Black tea	**0.45**	0.18	0.15	0.1	0.40 *	0.15	4.75	2.34
	Coca-Cola	0.33	0.15	**0.17**	0.15	0.50 *	0.2	3.42	1.5
	Red Bull	0.34	0.27	0.1	0.08	0.56	0.26	6.87	6.52
ClearCorrect	Coffee	**0.35**	0.15	0.17	0.08	0.49 *	0.17	4.28	1.46
	Black tea	0.17 *	0.13	**0.23**	0.12	0.60 *	0.15	2.99	1.07
	Coca-Cola	0.28	0.19	0.15 *	0.08	0.57 *	0.2	3.62	1.04
	Red Bull	0.15 *	0.12	0.06 *	0.04	**0.79**	0.15	4.86	2.18
Clear-A	Coffee	0.37 *	0.07	0.08 *	0.02	**0.54**	0.06	23.38	2.12
	Black tea	**0.57**	0.11	0.15	0.01	0.28 *	0.11	17.0	1.93
	Coca-Cola	0.46	0.21	**0.17**	0.17	0.37 *	0.18	4.36	2.93
	Red Bull	0.49	0.22	0.07 *	0.03	0.44	0.22	4.92	2.81
Tera Harz TC85	Coffee	0.35 *	0.08	0.11	0.02	**0.54**	0.08	26.38	2.39
	Black tea	0.44 *	0.06	**0.16**	0.02	0.40 *	0.05	27.04	1.58
	Coca-Cola	**0.76**	0.11	0.08	0.06	0.16 *	0.11	5.49	2.05
	Red Bull	0.52 *	0.32	0.1	0.19	0.38 *	0.24	3.73	1.92

Asterisks (*) indicate statistically significant differences between the highest ratio (bolded number) in the group, highlighting the dominant factor contributing to color change for each beverage–brand combination.

**Table 6:** Color change rating among brands and beverages.

Brand	Beverage	Mean
Invisalign	Coffee	18.45
	Black tea	4.37
	Coca-Cola	3.14
	Red Bull	6.32
ClearCorrect	Coffee	3.94
	Black tea	2.75
	Coca-Cola	3.33
	Red Bull	4.47
Clear-A	Coffee	21.51
	Black tea	15.64
	Coca-Cola	4.01
	Red Bull	4.53
Tera Harz TC 85	Coffee	24.27
	Black tea	24.87
	Coca-Cola	5.05
	Red Bull	3.43

## Data Availability

Data are contained within the article.
